# Succession of medico-legal important flesh flies (Diptera: Sarcophagidae) in the temporal gradient of pig decomposition in the Brazilian Cerrado

**DOI:** 10.1038/s41598-024-58898-8

**Published:** 2024-04-08

**Authors:** José Orlando de Almeida Silva, Fernando da Silva Carvalho-Filho, Leandro Schlemmer Brasil

**Affiliations:** 1 Programa de Pós-Graduação Em Zoologia (PPGZOOL), Instituto de Ciências Biológicas, Universidade Federal Do Pará (UFPA), Belém, PA Brazil; 2 Curso de Licenciatura Interdisciplinar em Ciências Naturais/Biologia (LCN/Biologia), Centro de Ciências de Codó (CCCO), Universidade Federal do Maranhão (UFMA), Codó, MA Brazil; 3 Laboratório de Entomologia, Programa de Pós-Graduação Em Zoologia (PPGZOOL), Departamento de Zoologia, Museu Paraense Emílio Goeldi (MPEG), Belém, PA Brazil; 4 Instituto de Ciências Biológicas e da Saúde (ICBS), Universidade Federal de Mato Grosso (UFMT), Pontal Do Araguaia, MT Brazil

**Keywords:** Ecology, Zoology

## Abstract

Flies belonging to the Sarcophagidae family play a significant role in forensic investigations by aiding in the estimation of post-mortem interval through the assessment of the developmental time of their immature stages and/or the succession patterns of species on carcasses and cadavers. In this regard, this study aimed to investigate the ecological succession of these flies on pig carcasses within Cerrado of Brazil. The progression of fly succession was examined over a 10-day period using the Threshold Indicator Taxa Analysis (TITAN) approach. Six Z + species (*Oxysarcodexia thornax* (Walker), *Peckia* (*Sarcodexia*) *lambens* (Wiedemann), *Peckia* (*Euboettcheria*) *collusor* (Curran & Walley), *Ravinia belforti* (Prado & Fonseca), *Tricharaea* (*Sarcophagula*) *canuta* (Wulp) and *Tricharaea* (*Sarcophagula*) *occidua* (Fabricius)) were identified, with change points ranging from 2.5 to 3 days during the dry season and 2.5 to 5.5 days during the rainy season. Two Z−  species (*Dexosarcophaga carvalhoi* (Lopes) and *Peckia* (*Sarcodexia*) *tridentata* (Hall)) were present, with a change point of 6.5 days during the rainy season. This study provides a continuous analysis of the temporal succession of flies, enabling an investigation into species progression based on their change points and directions (Z + and Z− ), thereby offering methodological advancements to avoid the arbitrary categorization of inherently continuous data.

## Introduction

In Brazil, particularly in the Northeast region, the occurrence of criminal incidents involving unresolved deaths, where suspects are either charged or acquitted, is substantial. This situation has sparked significant concern within the population^1^. In the state of Maranhão, which ranks as the second-largest state in terms of territorial extent in the Northeast region (encompassing 329,651.496 square kilometers, spanning 217 municipalities, and hosting an approximate population of 7,153,262 individuals), as well as the eighth largest state among the 26 states and the Federal District^2^, the homicide rate reached 24.1 deaths per 100,000 inhabitants in 2019. This figure surpasses the corresponding rates observed in numerous other states across the country^1^. Remarkably, a considerable portion of these homicides (80 cases) remained categorized as of undetermined cause, leading to a lack of judicial resolution due to insufficient evidence and a scarcity of tools capable of aiding in the resolution of these crimes^1^. Thus, the use of forensic entomology can contribute valuable information about the biology and ecological succession of flies (Diptera), beetles (Coleoptera) and other cadaveric insects, to assist police expertise as evidence in solving crimes involving homicides^3,4^.

In this context, it is of paramount importance to highlight the significant role of flesh flies, a member of the Sarcophagidae family (Diptera), as tool that can be useful in forensic studies within the medico-legal domain. This contribution is particularly notable in estimating the post-mortem interval in homicide investigations^3,4^. This phenomenon stems from the life cycle of flesh flies, which encompasses several developmental stages, commencing with the first instar larva and progressing through the second and third instars, ultimately culminating in the pupal phase. These immature forms primarily develop within carcasses and decomposing cadavers until attaining adult stage^3–6^. Multiple investigations have demonstrated that specific species of adult flesh flies appear during distinct phases of the decomposition process, specially in the early, intermediate, and advanced stages^3,4,7–9^.

In order to facilitate forensic assessments involving human remains using the aid of flesh flies, preliminary investigations are usually undertaken. These initial inquiries commonly employ carcass models of alternative vertebrate species, such as pigs, rats, rabbits, cats, dogs, among others, to acquire insights into the succession pattern and potential overlap of both adult and immature species across the process of cadaver decomposition. Pig carcasses are the most used in forensic studies, because they have some similarities with humans in size (amount of biomass), integument, proportion and distribution of hair, size of the rib cage, specificities of internal organs and also similarity of the fauna associated with decomposition^3,4,7–15^. These investigations require diverse environmental contexts, encompassing various seasons and regions characterized by distinct land use and coverage, including urban, rural, or natural vegetation areas. This approach allows for data extrapolation in order to establish the chronology of cadaver decomposition^3,4,9,14,16^.

In particular, when working with areas characterized by native vegetation, distinct biomes, or varying plant formations, there exists a notable diversity in the composition of Sarcophagidae species and the surrounding environmental conditions^7,9,10,14,17–19^. Consequently, it becomes imperative to investigate these contexts separately in order to enhance the accuracy of extrapolating previous knowledge to cadavers. In the Brazilian context, the limited studies available on flesh fly succession in pig carcasses predominantly focus on the Central-West region, with a particular emphasis on the Southeast region, since there is a greater number of researchers conducting studies for forensic purposes^10,12,14,20–22^. There are only a few studies conducted in Cerrado regions^10,14,21,23^, and these have been particularly scarce for the Cerrado of Maranhão, situated in the Northeastern region of the country. Within this specific Cerrado environment in Maranhão, only a study has focused on making an inventory and description of the succession patterns of flesh fly communities across different stages of pig carcass decomposition. Nevertheless, a comprehensive and analytical exploration of this ecological succession process has yet to be conducted^19^. This gap in the literature is especially significant, particularly since this dearth of understanding aligns with one of the most violent regions characterized by a low efficacy in solving homicides^1^. Consequently, it is of utmost importance for further studies involving decomposing carcasses to be conducted in this region, allowing the accumulated data on flesh flies to make a substantial contribution to the field of forensic science. In addition, in the face of a large biodiversity, detecting the pattern of change for a smaller number of species allows for greater agility in investigations and a reduction in the time to obtain answers, which is a great advance.

With the objective of elucidating the ecological succession dynamics of flesh flies for forensic applications, the present study was conducted to investigate the temporal progression of these flies throughout different stages of pig carcass decomposition within the Cerrado regions of Northeastern Brazil.

## Methods

### Experimental design

**Figure 1:**
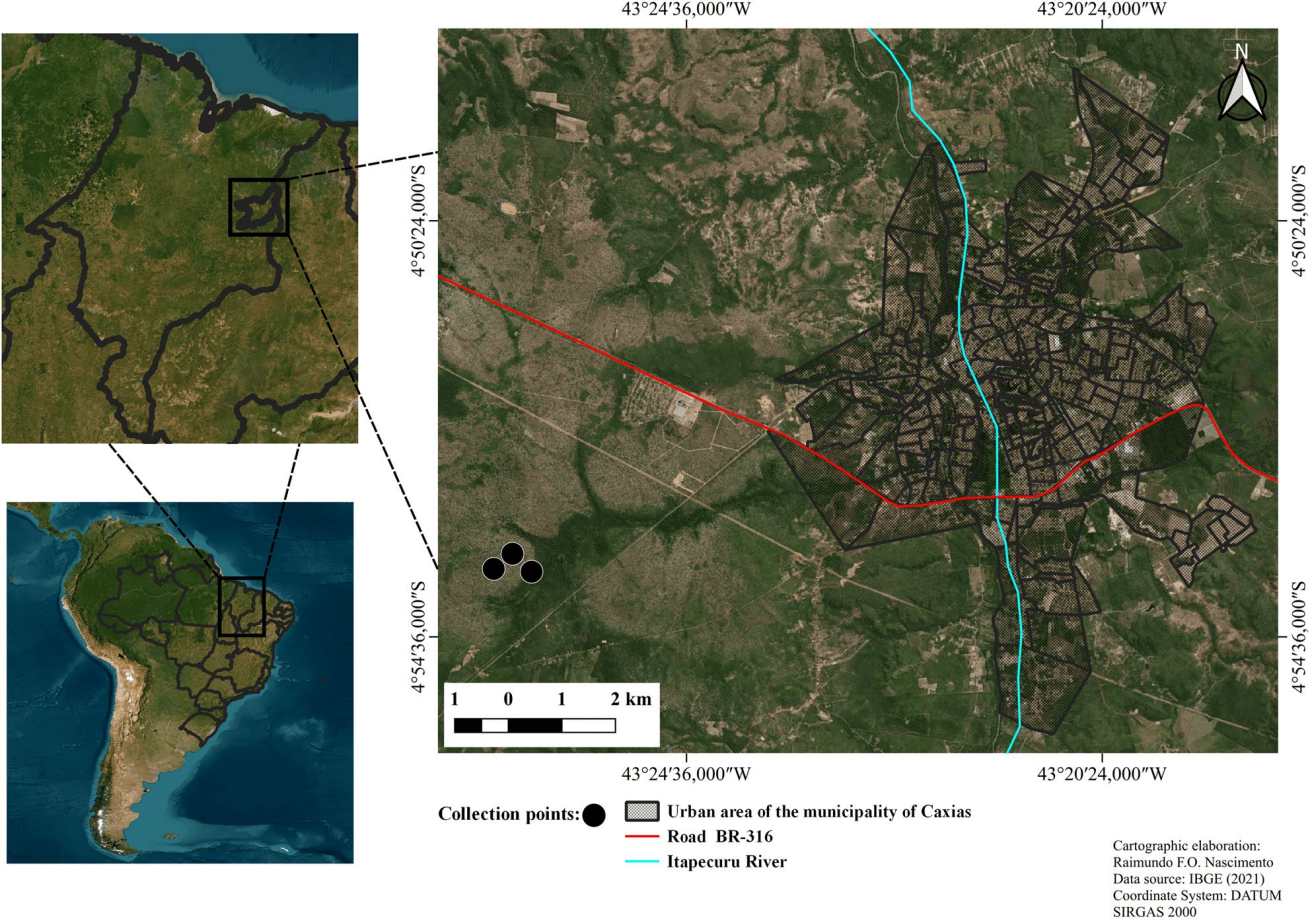
Collection points of flesh flies (Diptera, Sarcophagidae) on pig carcasses in the Cerrado areas of the Inhamum Municipal Environmental Protection Area, municipality of Caxias, state of Maranhão, Brazil. Source: IBGE^25^ (adapted by R.F.O. Nascimento 2022).

The research was conducted in Cerrado areas, which resembles a savanna ecosystem, situated in the eastern region of the state of Maranhão, located in Northeastern Brazil. These areas are specifically situated within the municipality of Caxias and fall under the jurisdiction of an established environmental protection zone known as the Municipal Environmental Protection Area of Inhamum (*APA do Inhamum*), previously referred to as the Inhamum Ecological Reserve (REI). Geographically, the area is positioned at coordinates 04°53′54.8′′S and 43°26′32.9′′W. The study site is traversed by the MA-127 highway, connecting Caxias to São João do Soter (Fig. 1). Following the Köppen climate classification system for Brazil as outlined by Alvares et al.^24^, the study regions are situated within zones classified as having a Tropical Aw climate—characterized by a dry winter, annual total precipitation ranging between 1300 and 1600 mm, and an average annual temperature surpassing 26 °C.

Two distinct experiments were undertaken in this study. The first one was performed during the dry season, during the months of July and August in 2010. This period exhibited minimal precipitation, with a recorded value of 1.7 mm. During this time frame, average temperature and relative humidity stood at 27.5 °C and 71%, respectively. The second experiment was conducted in the rainy season, during the months of March and April in 2011. This season was characterized by a notable precipitation total of 93.8 mm, along with average temperature and relative humidity values of 27 °C and 82%, respectively. Detailed information concerning the study locations has been outlined by Silva et al.^19^. It is noteworthy to mention that *APA do Inhamum*, according to local news sources, has been sporadically utilized for the disposal of cadavers (Attention, sensitive content: https://youtu.be/ScpUnkv342c; https://www.youtube.com/watch?v=DBQ778L71N4)^26–28^.

For experimental purposes, a total of six pigs carcasses (*Sus scrofa* Linnaeus; Artiodactyla: Suidae), each weighing 12 kg, were employed as animal models during both the dry and rainy seasons. The carcasses were obtained from a slaughterhouse. Following the ethical guidelines stipulated by the Ethics Commission on Animal Use (CEUA), the pigs were humanely euthanized on-site through a shot to the anterior part of the head, without suffering or pain to the animals, and without the use of chemical products to euthanasia. Subsequently, to prevent disturbance by scavenging vertebrates and to maintain the integrity of the carcasses, the pigs were placed within metal cages (110 × 85 × 85 cm; mesh size 3 × 3 cm). These cages were strategically positioned beneath tree canopies in shaded areas, with an approximate separation of 500 m between them. The present study was conducted with authorization from the Municipal Department of Environment and Preservation of Natural Resources of Caxias, MA (SEMUMA).

A suspended trap (measuring 150 × 160 cm), adapted from Rafael and Gorayeb^29^, was placed above each cage. This trap effectively covered over half of the upper section of the cage. The base of the trap was positioned 30 cm above the ground to facilitate the entry and capture of flies attracted to the pig carcasses. The flies captured by the collection cup, equipped with a strip of K-Othrine, a liquid insecticide commonly used for the eradication of predator insects like ants (Hymenoptera: Formicidae), were collected daily between 7 and 10 AM. Flies that were still alive were carefully transferred using an entomological net into a vial containing ethyl acetate for euthanization. The fly collection was authorized by the Biodiversity Authorization and Information System (SISBIO; License No. 12417).

The collected flies were preserved in 100 ml vials, meticulously labeled with information on the collection location, date, collector's name, and the specific pig carcass source. These vials contained a solution of 92.8% ethanol and were then deposited within the Zoological Collection of Maranhão (CZMA) at the Caxias Campus of the State University of Maranhão (UEMA) in the municipality of Caxias, located in the state of Maranhão, Northeastern Brazil. The male specimens underwent identification at the Laboratory of Invertebrate Studies (LEI) at UEMA. This process involved employing a stereomicroscope and entomological forceps, utilizing dichotomous keys, revisionary studies, and species descriptions from various sources, such as articles^30–47^, book chapters^48^, and theses^49,50^. Some specimens were also identified through comparisons with voucher material from CZMA and the Entomological Collection of the Emílio Goeldi Museum (MPEG) in Belém, Pará, Northern Brazil. Identification predominantly involved the examination of male genitalia, facilitated by careful manipulation using entomological forceps and pins within a petri dish containing alcohol. However, female specimens were not identified due to the absence of available literature to support such identifications.

After identification, specimens belonging to the same species were meticulously placed in distinct vials, each adequately labeled with sizes proportionate to the quantity of specimens. The voucher material resulting from this research is housed in the collections of both CZMA and MPEG. The comprehensive species list has been previously published by Silva et al.^19^.

### Data analysis

In the scope of the current study, each collection day for each pig carcass in both the dry and rainy seasons was treated as an individual sampling unit. Initially, a Procrustes analysis^51^ was conducted to evaluate the correspondence between the collected flesh fly communities in the two distinct seasons, i.e., dry and rainy. Based on the outcome, if the communities gathered during the dry and rainy seasons displayed substantial congruence, implying similarity across the year, the analyses would be pursued in a combined manner. Conversely, if the communities demonstrated incongruity or weak connection, indicating dissimilarity over the period, they would be independently scrutinized, considering the collections from both the dry and rainy seasons. The Procrustes analysis involves a comparison of two data matrices by aligning them using an ordination and rotational adjustment algorithm, generating "M12" values that indicate the extent of concordance between the datasets^51^.

A reduced “M12” value indicates a higher level of congruence between the data matrices. To perform the Procrustes analysis, the two matrices containing data of flesh fly species from the dry and rainy seasons underwent a logarithmic transformation using the formula log (x + 1). Subsequently, a Bray–Curtis distance matrix was computed, since the abundance data of the species were used. The logarithmic transformation was implemented to mitigate biases induced by non-normal data within the biological data matrix^52^. The Bray–Curtis distance matrix was employed for community ordination, facilitated by Principal Coordinates Analysis (PCoA)^52^. The PCoA axes were used as input for the Procrustes analysis, utilizing the Monte Carlo permutation test (9999 permutations). To establish congruence between the flesh fly communities across the dry and rainy seasons, two criteria were implemented. Initially, a significance level of 5% was applied; if the obtained result was not statistically significant (*p* > 0.05), the matrices would be deemed incongruent. Additionally, even when a significant relationship was present (*p* < 0.05), the M12 value needed to be < 0.30 to indicate a substantial level of congruence between the matrices.

To investigate alterations in the distribution patterns of visiting flesh flies species and to pinpoint their change points (cp) and distribution trends across the temporal gradient of pig carcass decomposition days, we employed the Threshold Indicator Taxa Analysis (TITAN). This analytical method identifies the change point (nCPA) of species abundance along an environmental gradient (in this case, the time of decomposition in days) and delineates the direction of this shift based on the scores derived from the Individual Indicator Value index (*IndVal*). Should a species exhibit heightened occurrence and increased abundance from the point of change towards the advanced stages of the carcass decomposition gradient, it is categorized as “Z + ”; conversely, if there is a surge in occurrence and abundance from the point of change towards the initial phases of the carcass decomposition gradient, it falls under the “Z− ” category^53,54^.

The criteria employed to designate a species as Z + or Z−  encompass purity and reliability levels of ≥ 90%, along with a *p*-value ≤ 0.05. These statistical values are ascertained through IndVal scores at each change point via bootstrap and permutation procedures. Less common species were omitted from TITAN analysis due to insufficient occurrences along the gradient to qualify as Z + or Z−  indicators. Consequently, solely species exhibiting a minimum of three consecutive days of occurrence, without alternation, and present in at least one carcass, as well as species with more than five collected specimens were considered for inclusion in the TITAN analysis^53,54^.

The analyses were carried out using the R software^55^. Principal Coordinates Analysis (PCoA) was performed using the vegdist function from the Vegan package^56^, and the cmdscale function from the Stats package^55^. Procrustes analysis was conducted using the protest function from the vegan package^56^. The TITAN analysis was executed using the TITAN function from the TITAN2 package^53^.

## Results

**Figure 2:**
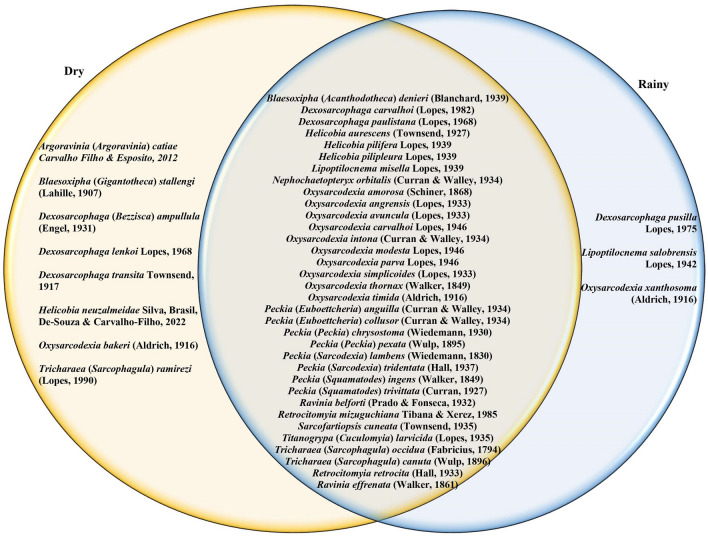
Sampling of flesh fly species across seasons, with identification of exclusive species and those shared between seasons in the Cerrado areas in Northeastern Brazil.

Throughout the duration of the carcass decomposition period, which spans approximately 10 days, a total of 10,819 flesh fly specimens were collected. Of these specimens, 40.07% were collected during the dry season, while the remaining 59.93% were gathered during the rainy season. Among the collected specimens, a total of 45 species were identified, with 42 species recorded during the dry season and 37 species during the rainy season. Thirty-four species were common in both seasons (Fig. 2; Supplementary Tables S1 and S2 online).

**Figure 3:**
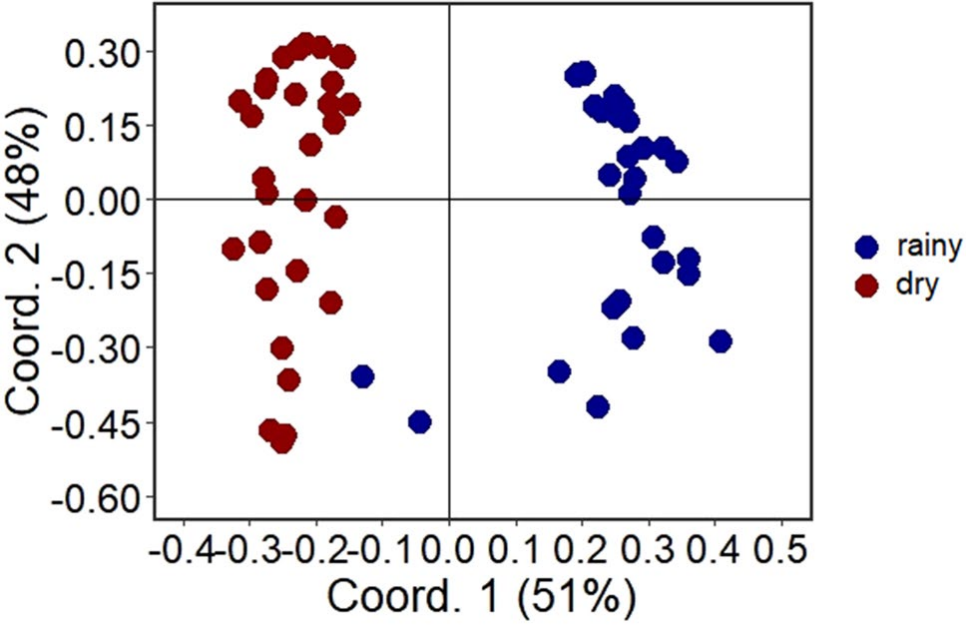
Multivariate ordination of matrices containing data on flesh flies collected in pig carcasses in Cerrado areas of Northeastern Brazil during dry and rainy seasons.

The congruence between the flesh fly communities during the dry and rainy seasons was found to be weak (M12 = 0.350, R = 0.807, *p* = 0.001; Fig. 3). Additionally, it was qualitatively noted that among the 45 sampled species, 11 species (24%), were exclusively present during either the dry or rainy season (Fig. 2; Supplementary Tables S1 and S2 online). Consequently, in light of this outcome, the analysis of flesh fly communities was performed separately for each respective season throughout the entirety of this study.

**Figure 4:**
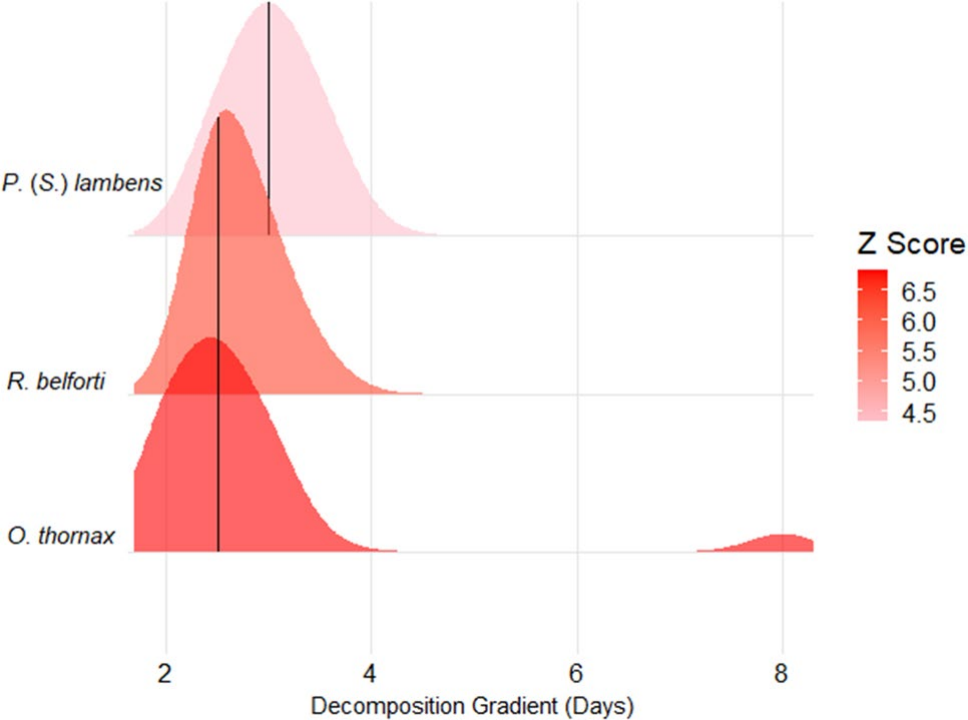
Change points and distribution of flesh fly species with positive responses to the days of decomposition gradient of pig carcasses in Cerrado areas during the dry season in Northeast Brazil. Z Score = score = level of association of species to the temporal gradient of days of decomposition of pigs carcasses; the greater the association of the species with the gradient, the higher its score value and the stronger the coloration pattern on the graph for each species; while the lower the association of the species with the gradient, the lower its score value and the weaker the coloration pattern on the graph for each species. *O*. = *Oxysarcodexia*; *P*. (*S*.) = *Peckia* (*Sarcodexia*); *R*. = *Ravinia*.

Nine indicator species were identified using TITAN as representative of carcass decomposition time, comprising three species during the dry season and six species during the rainy season. Specifically, *Oxysarcodexia thornax* (Walker, 1849) (Change Point: cp = 2.5 days of carcass decomposition), *Peckia* (*Sarcodexia*) *lambens* (Wiedemann, 1830) (cp = 3 days), and *Ravinia belforti* (Prado & Fonseca, 1932) (cp = 2.5 days) were categorized as Z + , signifying an augmentation in occurrence and abundance during the intermediate decomposition phases (Table 1 and Supplementary Table S3 online; Fig. 4). No Z−  indicator species were found during the dry season.

**Table 1:** Change points (zenv.cp = cp) and direction of response of flesh fly species (maxgrp) to the days of decomposition gradient of pig carcasses in Cerrado areas during the dry and rainy seasons in Northeastern Brazil.

Season	Species	zenv.cp	maxgrp	IndVal	obsiv.prob	zscore	purity	reliability
Dry	*Oxysarcodexia thornax* (Walker, 1849)	2.5	Z +	89.25	0.001	6.76	0.92	1.00
	*Peckia* (*Sarcodexia*) *lambens* (Wiedemann, 1830)	3.0	Z +	82.01	0.001	4.41	1.00	0.98
	*Ravinia belforti* (Prado & Fonseca, 1932)	2.5	Z +	92.19	0.001	6.18	0.98	0.98
Rainy	*Dexosarcophaga carvalhoi* (Lopes, 1980)	6.5	Z−	64.91	0.006	3.25	0.98	0.98
	*Peckia* (*Euboettcheria*) *collusor* (Curran & Walley, 1934)	2.5	Z +	90.43	0.001	6.23	0.92	1
	*Peckia* (*Sarcodexia*) *tridentata* (Hall, 1937)	6.5	Z−	52.94	0.001	4.37	0.98	0.96
	*R. belforti*	4.0	Z +	90.72	0.001	6.81	1	1
	*Tricharaea* (*Sarcophagula*) *canuta* (Wulp, 1896)	5.5	Z +	59.48	0.008	3.51	0.98	0.92
	*Tricharaea* (*Sarcophagula*) *occidua* (Fabricius, 1794)	3.0	Z +	100.00	0.001	6.45	1	1

IndVal, Indicator Value; obsiv.prob, significance of the result (p); zscore, score, level of association of species to the temporal gradient of days of decomposition of pigs carcasses; purity, proportion of change point response directions (positive or negative) between bootstrap replicates that agree with the observed response; reliability, probability of getting an equal or greater IndVal based on random permutations of the data set; Z − , decrease in occurrence and abundance of the species from the change point; Z + , increase in occurrence and abundance of the species from the change point.

**Figure 5:**
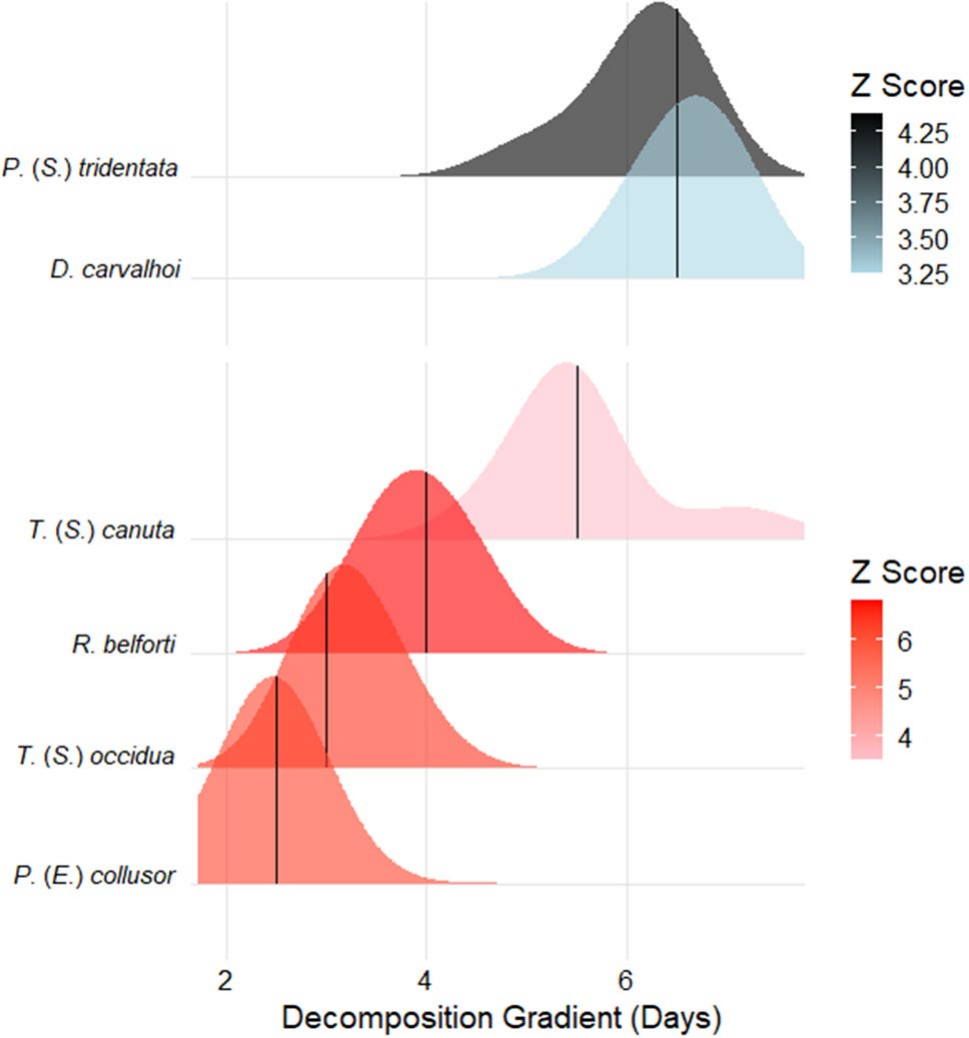
Change points and distribution of flesh fly species with positive (Z +) and negative (Z-) responses to the days of decomposition gradient of pig carcasses in areas of Cerrado during the rainy season in Northeastern Brazil. The red symbols, lines, or dots are the Z + species values; and the blue symbols, lines or dots are the Z−  species values. Z Score = score = level of association of species to the temporal gradient of days of decomposition of pigs carcasses; the greater the association of the species with the gradient, the higher its score value and the stronger the coloration pattern on the graph for each species; while the lower the association of the species with the gradient, the lower its score value and the weaker the coloration pattern on the graph for each species. *D*. = *Dexosarcophaga*;* P*. (*E*.) = *Peckia* (*Euboettcheria*); *P*. (*S*.) = *Peckia* (*Sarcodexia*); *R*. = *Ravinia*; *T*. (*S*.) = *Tricharaea* (*Sarcophagula*).

Throughout the rainy season, several species exhibited distinct temporal patterns. *Peckia* (*Euboettcheria*) *collusor* (Curran & Walley, 1934) (cp = 2.5 days), *R. belforti* (cp = 4 days), *Tricharaea* (*Sarcophagula*) *canuta* (Wulp, 1896) (cp = 5.5 days), and *Tricharaea* (*Sarcophagula*) *occidua* (Fabricius, 1794) (cp = 3 days) were categorized as Z + , showing an increase in occurrence and abundance towards the intermediate stages of decomposition. Conversely, *Dexosarcophaga carvalhoi* (Lopes, 1980) (cp = 6.5 days) and *Peckia* (*Sarcodexia*) *tridentata* (Hall, 1937) (cp = 6.5 days) were classified as Z− , demonstrating increased occurrence and abundance prior to the change point, namely towards the earlier stages of decomposition (Table 1 and Supplementary Table S4 online; Fig. 5).

## Discussion

This study delved into the succession dynamics of flesh flies on pig carcasses, highlighting distinct sets of indicator species for both the initial and advanced stages of decomposition. Given the marked climatic seasonality within the Maranhão Cerrado region, it became evident that these indicators must be identified separately for the dry and rainy seasons due to inherent variations in flesh fly communities across these periods. Season-specific climatic factors such as temperature, precipitation, and relative humidity play a pivotal role in shaping the cadaver's decay process^15^, thereby influencing the composition of flesh fly communities during each season. Additionally, the limited congruence observed in flesh fly communities across the two seasons underscores the necessity of employing tailored indicator species for accurate assessment in each distinct season.

Eight species of flesh flies (*D. carvalhoi*,* O. thornax*,* P.* (*E*.) *collusor*,* P.* (*S*.) *lambens*,* R. belforti*,* T*. (*S*.) *canuta*,* T*. (*S*.) *occidua*, and *P.* (*S*.) *tridentata*) were identified as indicators of ecological succession along the gradient of carcass decomposition days. These species, except for *P*. (*S*.) *tridentata*, exhibit extensive distribution not only within various biomes and regions of Brazil, but also across different countries in the Americas^9,17–19,23,40,46,57–61^. This widespread distribution underscores their potential applicability across a diverse range of geographical areas. *Peckia* (*S*.) *tridentata* has been recorded in the two largest biomes of the Neotropical region, namely the Cerrado and the Amazon Rainforest^19,23,40,57,59–61^. This observation implies a more limited potential for its role as an indicator within these particular biomes.

The flesh flies were observed throughout the entire process of carcass decomposition, from the beginning until complete skeletonization^19^. However, only a select few species were identified as being closely associated with specific stages of decomposition. In total, five Z + species were identified, all belonging to the genera *Oxysarcodexia* Townsend, 1917, *Peckia* Robineau-Desvoidy, 1830, *Ravinia* Robineau-Desvoidy, 1863, and *Tricharaea* Thomson, 1869. These genera encompass numerous species of recognized forensic significance^4,9,10,19,21,23,40,62^. Notably, these species exhibited change points and increased abundance values during the initial stages of the carcass decomposition gradient. This occurred within only 2.5 days for the species *O. thornax*, *R. belforti* (in the dry season), and *P*. (*E*.) *collusor* (in the rainy season); within 3 days for* P*. (*S.*) *lambens* (in the dry season) and *T*. (*S*.) *occidua* (in the rainy season); and within 4 days for *R. belforti* (in the rainy season).

The identification of the aforementioned species during the initial stage of the decomposition process, spanning from 2 to 3 days, aligns with the phase characterized by carcass swelling and the noticeable distension and separation of the legs^15,63^. By the fourth day, the swelling has subsided, and subtle cracks begin to form on the body. Additionally, this period marks the initial appearance of numerous larvae on the carcass^63^.

Typically, on the initial day of decomposition, carbon dioxide, a non-flammable gas, is released due to the activity of aerobic bacteria. Subsequently, between the second and fourth days of decomposition, flammable gases such as hydrogen and hydrocarbons emerge, resulting from the combined metabolic activities of both aerobic and anaerobic bacteria^3^. Consequently, the established change point thresholds for these species substantiate their responsiveness to the morphophysiological alterations inherent to the process of pig carcass decomposition.

*Ravinia belforti* was the only species displaying increased abundance values beyond the change point in both the dry and rainy seasons, making this observation particularly noteworthy. This finding holds substantial implications, as *R. belforti* could potentially serve as an indicator for estimating the post-mortem interval of cadavers located in Cerrado areas during both climatic periods. However, a distinction existed in the change point days for *R. belforti* between the two seasons. In the rainy season, the change point occurred on the fourth day, whereas in the dry season, it manifested at 2.5 days. This variation likely stems from differences in temperature, precipitation, and relative humidity experienced during these seasons, thereby influencing the physicochemical transformations inherent to carcass decomposition. Hence, in the dry season, the carcasses provided conditions conducive to the establishment of this species earlier than in the rainy season^22^.

Among the Z + species, *T*. (*S*.) *canuta* exhibited an increase in its abundance values only from 5.5 days onward, a pattern observed during the intermediate phase of carcass decomposition. This specific behavior could potentially be associated to an affinity with the presence of non-flammable gases, such as ammonia and nitrogen, which start to accumulate from the fifth day of decomposition^3^. On this day, noticeable changes were observed in the carcasses, including a visible reduction in body mass compared to preceding days, the appearance of wrinkles in the ventral region, and the emergence of fungal growth. Furthermore, the skeletal components of the body, such as the snout and limbs, began to be exposed.

The Z−  species identified during the rainy season, categorized under the genera *Dexosarcophaga*, Townsend, 1917 (*D*. *carvalhoi*) and *Peckia* (*P*. (*S*.) *tridentata*), exhibited their highest abundance values when the carcasses had reached an advanced stage of decomposition, with only 1.5 days remaining until they reached the skeletal stage^3,63^. Among these species, *P.* (*S*.) *tridentata* was the sole representative within the three *Peckia* species displaying the Z−  indicator trait. This distinct behavior in comparison to other species within the same genus can be attributed to significant variations in the immature and adult life history of *Peckia* species. These variations encompass diverse roles, spanning from sarcosaprophages, coprophages, frugivores, omnivores, to parasites of both vertebrates and invertebrates^9,18,19,40,58^.

Six out of the eight indicator species identified in this study are commonly found and have been observed in forensic studies involving pig carcasses within Cerrado areas of the Central-West and Southeast regions of Brazil (*D. carvalhoi*, *O. thornax*,* P*. (*E*.) *collusor*,* P*. (*S*.) *lambens*, *R. belforti*, *and T*. (*S*.) *occidua*)^14,21^. Both Barros et al.^21^ and Paseto et al.^14^ collected specimens of these species from carcasses at comparable stages of decomposition to those observed in the present study, with similar change points and trends in increased abundances. This congruence reinforces our findings and underscores the robust potential of these species as reliable forensic indicators for the Brazilian Cerrado ecosystem.

From an ecological perspective, the presence of a species at a specific place and period of the decomposition process adheres to the principles outlined in the Niche Theory, which underscores the association between species distribution and the availability of conditions and resources aligned with their physiological needs^64^. In addition, environmental, spatial and/or temporal heterogeneity enables the coexistence of sarcosaprophagous insect species in local communities, visitors and/or colonizers of carcasses and corpses, being mediated by interactions such as predation, parasitism, aggregation, intra and interspecific competition and dispersal capacity of inferior competitors^65^. Consequently, it is reasonable to anticipate that species to be present and exhibit higher abundance when conditions and resources closely align with their optimal requirements for establishment and development^66^. These conditions and resources tend to transition gradually rather than abruptly across time or space^67^. As a result, analyzing such data in a continuous manner is most suitable, minimizing the loss of information about the ecological succession of fly species in the process of decomposing carcasses and cadavers^52,67^. Thus, the adoption of methodologies like TITAN for the study of flesh fly species on pig carcasses is expected to represent the natural fluctuations of these species more accurately across temporal gradients of decomposition compared to analyses based on predefined temporal stages.

Hence, this study undertook a comprehensive examination of the ecological succession of flesh fly species (both Z + and Z− ) across the temporal gradient of pig carcass decomposition during both the dry and rainy seasons within the Cerrado biome of Northeastern Brazil. The Z + species demonstrated unique change points, indicating a substantial surge in their prevalence and abundance during the initial phases of decomposition. Conversely, the Z−  species exhibited later change points, showcasing heightened occurrences and levels of abundance in the later stages of decomposition. Considering the body proportions of the carcasses analyzed in this study in relation to the cadavers and taking into considering environmental factors, such as climate and phytophysiognomy, which vary greatly between regions and environments, which can alter the ecological succession pattern of the flesh fly species in the temporal gradient of carcass decomposition, we propose the combined utilization of the identified species to enhance the estimation of post-mortem interval in forensic studies.

Within this context, noteworthy among them are the Z + species *O. thornax*, *R. belforti,* and* P*. (*S*.) *lambens*, exhibiting potential to estimate the early stages (1–4 days) of post-mortem interval during the dry season. During the rainy season, three Z + species (*P*. (*E*.) *collusor*,* T*. (*S*.) *occidua*, and* R*. *belforti*) could be used for estimating the initial days (1–4 days); whereas two Z + species (*R*. *belforti* and* T*. (*S*.) *canuta*), along with two Z−  species (*P*. (*S*.) *tridentata* and* D*. *carvalhoi*), demonstrate potential to estimate it in intermediate days (4–6 days). Finally, this assemblage of species, excluding *R*. *belforti*, holds utility in estimating the advanced stages (from 6 days onwards). In general, the information provided by the present study confirms the existence of an ecological succession of flesh fly species along the gradient of days of decomposition of pig carcasses in Cerrado areas in Northeast Brazil. These data are of great importance for forensic entomology in the medico-legal area, as they can be used as a complement by forensic experts in the elucidation of cases involving the estimation of the post-mortem interval of cadavers.

### Supplementary Information

Supplementary Information.

## Data Availability

The authors declare that all data supporting the results of this study are available in the article.
