# Supplementary material for: Succession of medico-legal important flesh flies (Diptera: Sarcophagidae) in the temporal gradient of pig decomposition in the Brazilian Cerrado
Source: Sci Rep. 2024 Apr 8;14:8218. doi: 10.1038/s41598-024-58898-8 (PMC11001974; doi:10.1038/s41598-024-58898-8)
Supplement: Supplementary file 1 — Supplementary Information. [file 41598_2024_58898_MOESM1_ESM.pdf]

Succession of medico-legal important flesh flies (Diptera: Sarcophagidae) in the temporal gradient of pig decomposition in the Brazilian Cerrado

José Orlando de Almeida Silva, Fernando da Silva Carvalho-Filho & Leandro Schlemmer Brasil

Supplementary Table

Supplementary Table S1. Species of flesh flies (Diptera: Sarcophagidae) and abundance of male specimens distributed along the gradient of decomposition days of pig carcasses in the dry season of the year in Cerrado areas in the Municipal Environmental Protection Area of Inhamum, Caxias, Maranhão, Brazil.

Supplementary Table S2. Species of flesh flies (Diptera: Sarcophagidae) and abundance of male specimens distributed along the gradient of decomposition days of pig carcasses in the rainy season of the year in Cerrado areas in the Municipal Environmental Protection Area of Inhamum, Caxias, Maranhão, Brazil.

Supplementary Table S3. Change points (zenv.cp = cp) and direction of response of flesh fly species (maxgrp) to the days of decomposition gradient of pig carcasses in Cerrado areas in the dry season in Northeastern Brazil.
Note: Highlighted in bold are species with purity (proportion of change point response directions (positive or negative) between bootstrap replications that agree with the observed response) and reliability (probability of obtaining an equal or greater IndVal based on random permutations of the data set) greater than 90% and p-values < 0.05

(obsiv.prob). IndVal = Indicator Value; zscore = level of association of species with the temporal gradient of days of decomposition of pig carcasses; Z- = decrease in occurrence and abundance of the species from the change point; Z+ = increase in occurrence and abundance of the species from the change point.

Supplementary Table S4. Change points (zenv.cp = cp) and direction of response of flesh fly species (maxgrp) to the days of decomposition gradient of pig carcasses in Cerrado areas during the rainy season in Northeastern Brazil.

Note: Highlighted in bold are species with purity (proportion of change point response directions (positive or negative) between bootstrap replications that agree with the observed response) and reliability (probability of obtaining an equal or greater IndVal based on random permutations of the data) greater than 90% and p-values < 0.05 (obsiv.prob). IndVal = Indicator Value; zscore = level of association of species with the temporal gradient of days of decomposition of pig carcasses; Z- = decrease in occurrence and abundance of the species from the change point; Z+ = increase in occurrence and abundance of the species from the change point.

Supplementary Table S1.

Species	Day									
	1	2	3	4	5	6	7	8	9	10
Argoravinia (*Argoravinia*) *catiae*	0	0	5	0	1	3	0	0	0	0
Blaesoxipha (*Acanthodotheca*) *denieri*	0	0	0	2	1	0	0	1	1	0
Blaesoxipha (*Gigantotheca*) *stallengi*	0	0	3	0	0	0	0	0	0	0
Dexosarcophaga (*Bezzisca*) *ampullula*	0	0	0	0	1	0	1	0	0	0
Dexosarcophaga carvalhoi	5	19	165	135	63	53	35	22	12	8
Dexosarcophaga lenkoi	0	0	1	0	0	0	0	0	0	1
Dexosarcophaga paulistana	0	2	1	0	0	0	0	0	0	0
Dexosarcophaga transita	0	1	0	0	1	0	0	0	0	0
Helicobia aurescens	0	0	2	1	0	0	0	0	0	0
Helicobia pilifera	0	4	3	0	0	0	0	0	1	1
Helicobia pilipleura	0	0	0	0	1	0	0	1	0	0
Helicobia neuzalmeidae	0	1	0	0	0	0	0	0	0	0
Lipoptilocnema misella	0	0	0	0	0	0	1	1	1	0
Nephochaetopteryx orbitalis	0	0	5	0	0	0	2	0	0	0
Oxysarcodexia amorosa	0	3	39	38	13	7	11	4	5	5
Oxysarcodexia angrensis	0	3	10	9	9	3	3	1	3	1
Oxysarcodexia avuncula	1	7	20	20	6	6	6	8	3	3
Oxysarcodexia bakeri	0	0	0	0	0	0	1	0	0	0
Oxysarcodexia carvalhoi	0	5	35	19	14	7	9	4	1	4
Oxysarcodexia intona	0	0	0	1	1	1	1	0	0	0
Oxysarcodexia modesta	0	0	1	0	2	0	0	1	0	0
Oxysarcodexia parva	0	0	3	3	5	5	4	1	6	2
Oxysarcodexia simplicoides	0	0	4	2	2	1	2	0	2	0
Oxysarcodexia thornax	1	40	219	250	175	188	243	115	85	86
Oxysarcodexia timida	7	33	146	162	103	61	73	36	43	25
Peckia (*Euboettcheria*) *anguilla*	0	0	0	0	1	0	0	0	0	0
Peckia (*Euboettcheria*) *collusor*	0	23	167	210	107	71	50	56	53	25
Peckia (*Peckia*) *chrysostoma*	0	0	1	0	0	0	0	0	0	0
Peckia (*Peckia*) *pexata*	0	1	6	1	4	0	5	1	0	0
Peckia (*Sarcodexia*) *lambens*	0	1	4	14	8	13	11	8	8	12
Peckia (*Sarcodexia*) *tridentata*	0	0	2	2	1	0	2	2	0	0
Peckia (*Squamatodes*) *ingens*	0	0	1	1	1	1	1	1	1	4
Peckia (*Squamatodes*) *trivittata*	0	3	2	4	1	3	2	0	3	2
Ravinia belforti	1	4	33	42	23	25	40	24	28	21
Ravinia effrenata	0	0	0	2	0	0	0	0	0	0
Retrocitomyia mizuguchiana	0	1	1	0	2	2	0	0	0	0
Retrocitomyia retrocita	0	1	1	0	0	0	0	0	0	0
Sarcofartiopsis cuneata	0	0	0	0	2	0	0	0	0	0
Titanogrypa (*Cuculomyia*) *larvicida*	0	0	2	1	3	1	0	0	0	0
Tricharaea (*Sarcophagula*) *canuta*	0	10	1	4	4	2	2	0	0	1
Tricharaea (*Sarcophagula*) *occidua*	0	9	5	14	14	14	13	4	8	3
Tricharaea (*Sarcophagula*) *ramirezi*	0	0	5	3	1	2	0	0	0	0

Supplementary Table S2.

Species	Day										
	1	2	3	4	5	6	7	8	9	10	11
Blaesoxipha (*Acanthodotheca*) *denieri*	0	0	0	0	0	1	0	0	0	0	0
Dexosarcophaga carvalhoi	1	4	18	9	5	9	5	0	1	1	0
Dexosarcophaga paulistana	0	0	0	0	0	1	0	0	0	0	0
Dexosarcophaga pusilla	0	2	0	0	0	0	0	0	0	0	0
Helicobia aurescens	0	2	3	0	0	1	0	0	0	0	0
Helicobia pilifera	0	3	3	1	3	3	2	1	1	0	0
Helicobia pilipleura	0	7	8	2	2	2	2	1	1	1	0
Lipoptilocnema misella	0	0	2	2	2	1	1	1	0	0	0
Lipoptilocnema salobrensis	0	2	1	1	1	0	0	0	0	0	1
Nephochaetopteryx orbitalis	0	1	10	8	2	3	1	0	3	0	1
Oxysarcodexia amorosa	0	3	19	12	11	5	4	1	2	0	1
Oxysarcodexia angrensis	0	0	4	4	16	6	13	5	9	3	1
Oxysarcodexia avuncula	1	0	0	1	1	1	1	0	0	0	0
Oxysarcodexia carvalhoi	0	13	27	25	25	13	8	6	5	2	0
Oxysarcodexia intona	0	0	0	0	1	0	0	0	0	0	0
Oxysarcodexia modesta	0	0	1	0	5	1	2	1	1	1	0
Oxysarcodexia parva	0	1	1	1	1	1	3	2	1	0	1
Oxysarcodexia simplicoides	0	0	1	0	0	0	0	0	0	0	0
Oxysarcodexia thornax	8	29	150	184	186	185	112	60	38	19	8
Oxysarcodexia timida	0	7	46	82	87	92	52	26	14	3	8
Oxysarcodexia xanthosoma	0	0	0	0	0	0	1	0	0	0	0
Peckia (*Euboettcheria*) *anguilla*	0	1	4	3	2	2	0	4	0	1	0
Peckia (*Euboettcheria*) *collusor*	1	21	113	111	183	115	120	91	43	33	23
Peckia (*Peckia*) *chrysostoma*	0	1	2	4	8	3	5	1	1	0	0
Peckia (*Peckia*) *pexata*	0	4	53	43	58	45	17	16	7	2	1
Peckia (*Sarcodexia*) *lambens*	14	182	459	425	482	509	442	286	163	123	81
Peckia (*Sarcodexia*) *tridentata*	0	1	3	2	1	3	0	0	0	0	0
Peckia (*Squamatodes*) *ingens*	0	3	4	1	4	1	2	2	0	0	0
Peckia (*Squamatodes*) *trivittata*	0	3	15	11	2	2	4	2	1	0	0
Ravinia belforti	0	0	1	7	13	14	14	17	8	6	0
Ravinia effrenata	0	0	0	0	1	0	1	1	1	0	0
Retrocitomyia mizuguchiana	0	0	1	0	2	0	1	0	0	1	0
Retrocitomyia retrocita	0	0	1	0	0	0	1	1	1	0	0
Sarcofartiopsis cuneata	0	0	0	0	0	0	0	0	0	0	1
Titanogrypa (*Cuculomyia*) *larvicida*	0	0	0	0	0	1	0	0	0	0	0
Tricharaea (*Sarcophagula*) *canuta*	0	1	1	1	1	4	2	2	6	5	2
Tricharaea (*Sarcophagula*) *occidua*	0	0	13	14	21	40	22	28	15	11	4

Supplementary Table S3.

**Species**	**zenv.cp**	**maxgrp**	**IndVal**	**obsiv.prob**	**zscore**	**purity**	**reliability**
Dexosarcophaga carvalhoi	7.0	Z-	81.40	0.003	3.40	0.88	1.00
Oxysarcodexia amorosa	2.5	Z+	75.87	0.012	2.93	0.70	0.82
Oxysarcodexia angrensis	5.0	Z-	38.15	0.204	0.70	0.44	0.54
Oxysarcodexia avuncula	5.0	Z-	55.28	0.130	1.20	0.38	0.72
Oxysarcodexia carvalhoi	2.5	Z+	48.01	0.229	0.80	0.52	0.40
Oxysarcodexia parva	2.5	Z+	58.33	0.025	2.50	0.92	0.82
Oxysarcodexia simplicoides	2.5	Z+	41.67	0.137	1.45	0.62	0.52
O. thornax	**2.5**	**Z+**	**89.25**	**0.001**	**6.76**	**0.92**	**1.00**
Oxysarcodexia timida	2.0	Z+	84.71	0.003	4.20	0.58	1.00
Peckia (*Euboettcheria*) *collusor*	2.5	Z+	88.93	0.002	4.18	0.82	0.92
Peckia (*Peckia*) *pexata*	7.5	Z-	41.87	0.075	1.76	0.90	0.82
P. (S.) lambens	**3.0**	**Z+**	**82.01**	**0.001**	**4.41**	**1.00**	**0.98**
Peckia (*Sarcodexia*) *tridentata*	8.0	Z-	27.27	0.232	1.09	0.38	0.22
Peckia (*Squamatodes*) *ingens*	8.5	Z+	51.28	0.042	2.37	1.00	0.84
Peckia (*Squamatodes*) *trivittata*	8.5	Z+	38.10	0.306	0.37	0.60	0.34
R. belforti	**2.5**	**Z+**	**92.19**	**0.001**	**6.18**	**0.98**	**0.98**
Titanogrypa (*Cuculomyia*) *larvicida*	6.5	Z-	33.33	0.053	2.36	0.94	0.68
Tricharaea (*Sarcophagula*) *canuta*	7.5	Z-	34.59	0.175	1.20	0.70	0.36
Tricharaea (*Sarcophagula*) *occidua*	8.0	Z-	52.99	0.129	1.11	0.58	0.64
Tricharaea (*Sarcophagula*) *ramirezi*	6.5	Z-	27.78	0.111	1.81	0.92	0.60

Supplementary Table S4.

**Species**	**zenv.cp**	**maxgrp**	**IndVal**	**obsiv.prob**	**zscore**	**purity**	**reliability**
D. carvalhoi	**6.5**	**Z-**	**64.91**	**0.006**	**3.25**	**0.98**	**0.98**
Helicobia pilifera	9.0	Z-	48.00	0.058	1.60	0.60	0.60
Helicobia pilipleura	2.0	Z+	36.00	0.228	0.88	0.50	0.24
Lipoptilocnema misella	8.0	Z-	31.82	0.142	1.31	0.64	0.36
Lipoptilocnema salobrensis	5.0	Z-	33.36	0.061	2.62	0.84	0.54
Nephochaetopteryx orbitalis	2.0	Z+	52.00	0.052	1.61	0.50	0.64
O. amorosa	2.0	Z+	72.00	0.018	2.86	0.34	0.94
O. angrensis	4.0	Z+	48.23	0.033	2.26	0.98	0.68
O. avuncula	7.5	Z-	23.88	0.260	1.23	0.84	0.20
O. carvalhoi	9.0	Z-	73.94	0.222	2.56	0.80	0.98
Oxysarcodexia modesta	4.5	Z+	39.11	0.052	2.25	0.96	0.66
O. parva	6.0	Z+	33.39	0.159	1.11	0.74	0.40
O. thornax	2.5	Z+	86.42	0.001	4.70	0.80	1
O. timida	2.5	Z+	93.61	0.001	5.17	0.88	1
Peckia (*Euboettcheria*) *anguilla*	2.0	Z+	48.00	0.060	1.60	0.40	0.56
P.* (*E.*) *collusor	**2.5**	**Z+**	**90.43**	**0.001**	**6.23**	**0.92**	**1**
Peckia (*Peckia*) *chrysostoma*	9.0	Z-	60.00	0.052	2.09	0.58	0.88
P. (*P.*) *pexata*	2.5	Z+	93.80	0.001	5.11	0.78	1
P. (*S.*) *lambens*	2.0	Z+	81.44	0.001	5.85	0.82	1
P.* (*S.*) *tridentata	**6.5**	**Z-**	**52.94**	**0.001**	**4.37**	**0.98**	**0.96**
P. (*S.*) *ingens*	8.0	Z-	50.00	0.033	2.48	0.82	0.60
P. (*S.*) *trivittata*	8.0	Z-	52.13	0.049	1.83	0.60	0.74
R. belforti	**4.0**	**Z+**	**90.72**	**0.001**	**6.81**	**1**	**1**
T.* (*S.*) *canuta	**5.5**	**Z+**	**59.48**	**0.008**	**3.51**	**0.98**	**0.92**
T.* (*S.*) *occidua	**3.0**	**Z+**	**100.00**	**0.001**	**6.45**	**1**	**1**
